# Polymorphism analysis of estrogen receptor β Gene RsaI and AluI in girls with idiopathic central precocious puberty: investigating the relationship and implications for early risk prediction

**DOI:** 10.3389/fendo.2026.1809152

**Published:** 2026-06-17

**Authors:** Peipei Liu, Guoqing Dong, Mingzhu Li, Miao Huang, Xiyan Lu, Jianxu Li

**Affiliations:** Shenzhen Maternity and Child Healthcare Hospital, Women and Children’s Medical Center, Southern Medical University, Shenzhen, Guangdong, China

**Keywords:** allele frequency, estrogen receptor beta, genetic polymorphism, idiopathic central precocious puberty, RsaI

## Abstract

**Objective:**

To investigate the association between estrogen receptor β (*ERβ*) gene RsaI (rs1256049) and AluI (rs4986938) polymorphisms and the risk of idiopathic central precocious puberty (ICPP) in girls, and to provide potential molecular genetic references for early risk prediction and individualized management of ICPP.

**Methods:**

A total of 100 girls with ICPP from the Pediatric Endocrinology Clinic and Inpatient Ward of Shenzhen Maternity & Child Healthcare Hospital were enrolled, and 100 agematched healthy girls undergoing routine physical examinations during the same period served as controls. The RsaI and AluI polymorphisms of the *ERβ* gene were genotyped using polymerase chain reactionrestriction fragment length polymorphism (PCRRFLP). Genotype and allele frequencies were compared between the two groups, and haplotype analysis was performed to evaluate combined effects of the two loci.

**Results:**

1.For the *ERβ* RsaI polymorphism, significant differences were observed between the ICPP and control groups in both genotype distribution (*χ*^2^ = 5.96, *P* = 0.04) and R allele frequency (41.50% vs. 30.00%; *χ*^2^ = 4.77, *P* = 0.03). The R allele was associated with an increased risk of ICPP (odds ratio [OR] = 1.58, 95% confidence interval [CI]: 1.05–2.38, *P* < 0.05). 2.For the AluI polymorphism, no significant differences were found in either genotype distribution or A allele frequency between the two groups (*P* > 0.05). 3.Haplotype analysis of the combined RsaI and AluI polymorphisms showed a significant difference in overall haplotype distribution between the two groups (Fisher’s exact test, *P* = 0.02). The Rraa haplotype was more frequent in the ICPP group (39%) than in controls (31%).

**Conclusion:**

The *ERβ* gene RsaI polymorphism is significantly associated with the risk of ICPP in girls. The R allele may be a susceptibility factor, and carriers of the Rr genotype appear to have a higher risk of developing ICPP. The Rraa haplotype may represent a potential risk factor for ICPP. In contrast, the AluI polymorphism shows no significant association with ICPP. These findings suggest that *ERβ* RsaI genotyping and haplotype analysis could contribute to early risk assessment, although further multicenter studies with larger sample sizes are warranted to validate the results.

## 1 Introduction

Idiopathic central precocious puberty (ICPP) is a developmental disorder characterized by the premature appearance of secondary sexual characteristics. The etiology of ICPP in girls remains unclear and is likely attributed to genetic polymorphisms.And there are variations in genetic background (1, 2). The estrogen receptor (ER) plays a crucial role in the development of secondary sexual characteristics and the pubertal growth spurt in females (3–6). Studies have indicated that ERβ gene polymorphisms may influence bone metabolism and gonadal development by regulating estrogen signaling pathways. ERβ is highly expressed in bone cells, yet its specific function in these cells remains poorly understood (7, 8). Two common polymorphisms in the ERβ gene are situated in the ligand-binding domain of exon 5 (RsaI) and the 3′-untranslated region of exon 8 (AluI) (9). Different genotypes may determine inter-individual differences in ER expression levels and function, thus affecting the biological actions of estrogen (10, 11). A limited number of domestic studies have reported associations between ER polymorphisms and precocious puberty or advanced bone age (12, 13).

In this study, PCR-RFLP was utilized to identify RsaI and AluI polymorphisms within the ERβ gene among girls diagnosed with Idiopathic Central Precocious Puberty (ICPP), with the goal of investigating the potential association between these genetic variations and the development of ICPP.

## 2 Subjects and methods

### 2.1 Subjects

We selected 100 girls diagnosed with ICPP from the pediatric endocrinology outpatient and inpatient departments of Shenzhen Maternity and Child Healthcare Hospital. The age of initial diagnosis ranged from 4 to 9.2 years, with a mean age of (6.90 ± 1.38) years old. All patients underwent comprehensive assessments including medical history, physical examination, hormone level measurement, ultrasound of the uterus, ovaries, liver, and adrenal glands, bone age assessment, and pituitary MRI (plain scan + enhancement). They were confirmed to meet the diagnostic criteria for precocious puberty (14) through a gonadotropin-releasing hormone (GnRH) stimulation test using Gonadorelin.A control group of 100 healthy girls, randomly selected from the same hospital for health check-ups during the same period, was also included. These girls did not exhibit secondary sexual characteristics, had normal growth and development, and were free from chronic wasting diseases. Their ages ranged from 3.83 to 9 years, with a mean age of (6.62 ± 1.29) years. No significant age difference was observed between the case and control groups (*t* = 1.49, *P* = 0.14). All study participants provided informed consent, and the study was approved by the Ethics Committee of Shenzhen Maternity and Child Healthcare Hospital (Ethics Approval No.: SFYLS [2024]136).

Sample size justification: The sample size of 100 cases and 100 controls was determined based on a power calculation. Assuming an R allele frequency of 30% in the control group (based on preliminary local data) and an expected odds ratio of 1.8 for the association between the RsaI polymorphism and ICPP, with α = 0.05 and power = 0.80, the minimum required sample size was calculated to be 92 per group. Accounting for a potential 10% genotyping failure or dropout, we enrolled 100 participants per group. The calculation was performed using PASS software.

### 2.2 Sample collection

Three milliliters of venous blood were collected from each participant using sodium citrate anticoagulant and stored at −80 °C.

### 2.3 Experimental methods

#### 2.3.1 DNA extraction

DNA was extracted using an automated whole blood cell extraction kit (Xiamen Zhishan).

#### 2.3.2 Primer design and synthesis

Primers were synthesized by Shanghai Sangon Biological Engineering Technology & Services. The sequences were as follows: RsaI forward primer: 5’-TCTTGCTTTCCCCAGGCTTT-3’, reverse primer: 5’-ACCTGTCCAGAACAAGATCT-3’; AluI forward primer: 5’-TTTTTGTCC CCATAGTAACA-3’, reverse primer: 5’-AATGAGGGACCACACAGCA-3’.

#### 2.3.3 PCR amplification

PCR amplification was conducted using a Bio-Rad DNA Engine. The reaction system had a total volume of 50 µL, comprising 25 µL of PCR Master Mix (Taq DNA Polymerase, PCR Buffer, dNTP), 2 µL of each primer, 20 µL of deionized water, and 1 µL of DNA sample. The reaction conditions were as follows: pre-denaturation at 94 °C for 4 minutes, followed by 30 cycles of denaturation at 94 °C for 30 seconds, annealing at 60 °C for 30 seconds, and extension at 72 °C for 1 minute, concluding with a final extension at 72 °C for 10 minutes. PCR products were then analyzed using 2% agarose gel electrophoresis, and results were observed using a UV transilluminator (Model 752N).

#### 2.3.4 Restriction enzyme digestion analysis

For enzyme digestion, 10 µL of PCR product was mixed with 1 µL of RsaI and 1 µL of AluI (both from NEB, USA), and incubated at 37 °C for 2 hours. The resulting products were analyzed by 2% agarose gel electrophoresis, and results were observed using a UV transilluminator.

#### 2.3.5 Haplotype analysis

To explore the potential synergistic effects of RsaI and AluI loci, haplotype distribution was further analyzed to evaluate the impact of multi-gene joint effects on the risk of ICPP.

#### 2.3.6 Statistical methods

Statistical analyses were performed using SPSS 23.0 software. Hardy-Weinberg equilibrium was calculated to assess sample representativeness. Continuous variables were expressed as mean ± standard deviation (SD). The comparison of allele frequencies between genotype groups was conducted using the χ² test. Odds ratios (OR) and 95% confidence intervals (95% CI) were calculated to determine relative risk, with P<0.05 considered statistically significant.

## 3 Results

### 3.1 ERβ RsaI polymorphism analysis

Significant differences were found in the distributions of RsaI genotypes and R allele frequencies between the two groups (*P* < 0.05). The frequency of the R allele in the ICPP group was 41.50%, which was significantly higher than in the control group (30.00%), with an odds ratio (OR) of 1.58 (95% CI: 1.05–2.38, *P* < 0.05). Regarding genotype distribution, the Rr and rr genotypes were predominant in both the ICPP group (51% and 33%, respectively) and the control group (44% and 48%, respectively). See **Table 1**.

**Table 1:** Distribution of ERβ RsaI and AluI polymorphisms (n, %).

Group	ICPP group (n=100)	Control group (n=100)	χ²	*P*	OR (95% CI)
Genotype Frequency	RR	16 (16.00)	8 (8.00)	5.96	0.04
	Rr	51 (51.00)	44 (44.00)		
	rr	33 (33.00)	48 (48.00)		
Allele Frequency	R	83 (41.50)	60 (30.00)	4.77	0.03
	r	117 (58.50)	140 (70.00)		
Genotype Frequency	AA	3 (3.00)	1 (1.00)	2.89	0.24
	Aa	31 (31.00)	23 (23.00)		
	aa	66 (66.00)	76 (76.00)		
Allele Frequency	A	37 (18.50)	25 (12.50)	2.75	0.09
u	a	163 (81.50)	175 (87.50)		

### 3.2 ERβ AluI polymorphism analysis

No significant differences were observed in the distributions of AluI genotypes or A allele frequencies between the two groups *(P* > 0.05). The A allele frequency was 18.50% in the ICPP group and 12.50% in the control group, with an OR of 1.59 (95% CI: 0.92–2.76, *P* > 0.05). The aa genotype was the most common in both groups (66% in the ICPP group and 76% in the control group). See **Table 1**.

### 3.3 Combined analysis of ERβ RsaI and AluI polymorphisms

The distribution of nine haplotypes differed significantly between the two groups (P = 0.02), as determined by Fisher’s exact test. See **Table 2**.

**Table 2:** Combined haplotype analysis of ERβ RsaI and AluI polymorphisms (n, %).

Group	n	RsaI and AluI polymorphic haplotype								
		RRAA	RRAa	RRaa	RrAA	RrAa	Rraa	rrAA	rrAa	rraa
ICPP Group	100	1 (1)	8 (8)	7 (7)	1 (1)	11 (11)	39 (39)	1 (1)	12 (12)	20 (20)
Control Group	100	1 (1)	4 (4)	3 (3)	0 (0)	13 (13)	31 (31)	0 (0)	6 (6)	42 (42)
*P* value	0.02									

## 4 Discussion

Idiopathic central precocious puberty (ICPP) is a significant pediatric endocrine disorder caused by the premature activation of the hypothalamic-pituitary-gonadal axis, leading to early onset of secondary sexual characteristics in girls. Despite its clinical prevalence, the pathogenesis of ICPP remains incompletely understood, with genetic predispositions and molecular mechanisms being key areas of ongoing investigation. Notably, the estrogen receptor beta (ERβ), a nuclear hormone receptor involved in mediating estrogenic effects on reproductive tissue development and bone metabolism, has emerged as a candidate gene that influences pubertal timing. This may be related to the genetic regulatory mechanisms of estrogen, in addition to physiological fluctuations (15). Different ER genotypes may determine interindividual differences in ER expression and function, thereby influencing the biological effects of estrogen (8, 16, 17). However, the relationship between ERβ gene polymorphisms and susceptibility to ICPP has not been definitively established, with existing studies yielding inconsistent findings. Elucidating the genetic factors underlying ICPP is critical for improving early diagnosis and developing personalized therapeutic strategies, especially given the complex interplay between genetic variants and endocrine regulation in pubertal development.

Studies have shown a close relationship between pubertal development and ER (18). ER is a ligand-dependent transcription factor that regulates gene transcription through estrogen response elements (EREs), thereby mediating estrogen’s biological functions via gene regulation. However, dysregulation of ER signaling can lead to multiple disorders, including cancers, gynecological disorders, and neurological conditions (19–21). Classical ERs include ERα and ERβ, which are localized in the nucleus, exerting genomic regulatory effects by modulating specific target genes. ERβ is predominantly expressed in ovarian granulosa cells and is crucial for steroidogenesis, gonadotropin production, follicular development, and ovulation. Studies have shown that ERβ is highly expressed in the ovaries of mice during the estrous cycle and early pregnancy, with peak expression during the estrous phase, indicating its essential role in maintaining normal ovarian physiological functions (22). Guo demonstrated that ERβ mediates estrogen feedback regulation and anti-apoptotic functions in granulosa cells (17). Estrogen exerts its effects by binding to estrogen receptors ERα and ERβ, which are ligand-inducible transcription factors. Upon activation by estrogen, ERβ specifically binds to the promoter region of the CYP19A1 gene, thereby inducing its transcription. This process is part of the classical mechanism of estrogen action, where the receptor forms a dimer and interacts with the estrogen response elements (EREs) on the target gene’s promoter to modulate gene expression. This study provided important insights into steroid hormone regulation and female fertility enhancement. Asiamah found that the ERβ gene is involved in hormonal regulation during reproduction, enriching the estrogen signaling pathway related to reproductive performance (23). Recent research has highlighted the critical role of ERβ expression within hypothalamic GnRH neurons in orchestrating the pre-ovulatory gonadotropin surge, as detailed in the study by Eun B. Lee et al. Moreover, ERβ deficiency reduces pulsatile GnRH release, leading to delayed puberty in female ERβ-knockout mice (24). Baetens reported that ESR2 knockout mouse models resulted in infertility in both males and females without sex reversal (25). Other studies indicate that GnRH neurons primarily express ESR2 rather than ESR1 (26). Biason-Lauber also suggested that ESR2 may partially influence the hypothalamic– pituitary– gonadal axis and gonadotropin secretion. Loss-of-function mutations in ERβ may lead to testicular and ovarian dysgenesis, indicating that ESR2 is essential for gonadal determination in both sexes, while ESR1 alone is insufficient to drive gonadal development (27).

Recent studies have demonstrated significant associations between polymorphisms in estrogen receptor (ER) genes and the onset of precocious puberty, as evidenced by research conducted in Jiangxi province and other regions. Gene polymorphisms refer to the presence of multiple alleles at a single locus, which mainly include DNA sequence polymorphisms and length polymorphisms. DNA sequence polymorphisms are typically categorized as single nucleotide polymorphisms (SNPs) and restriction fragment length polymorphisms (RFLPs). Currently, the most studied polymorphisms in the ERβ gene are RsaI (rs1256049) and AluI (rs4986938). One study found no association between ERβ polymorphisms and age at menarche in patients with idiopathic scoliosis (28). Study suggested that the ERβ rs1256049 locus may be a susceptibility site for male infertility, while the AG genotype at rs4986938 might reduce the risk of male infertility (29). Reports on ER polymorphisms and central precocious puberty are limited and inconsistent. A study reported no significant association between PvuII and XbaI polymorphisms in the ERα gene and ICPP, which is consistent with findings from other studies that also found no significant correlation between these polymorphisms and the risk of ICPP (18, 30). Recent studies have reported associations between polymorphisms of the ERα gene and the onset of ICPP in girls (31, 32). However, studies on ERβ polymorphisms and precocious puberty are even rarer. Gu conducted a meta-analysis suggesting that the ERβ rs1256049 polymorphism may be a susceptibility factor for central precocious puberty (33). Ke JW reported that studies have shown an association between ERβ RsaI polymorphisms and the occurrence of precocious puberty in girls (12). Wang Xuewei also found that ERβ RsaI polymorphisms were associated with precocious puberty, but not AluI polymorphisms (34). The R allele was associated with a significantly increased risk of precocious puberty compared to the r allele, with individuals carrying the Rr genotype exhibiting the highest risk. In combined polymorphism analyses, Rraa and RrAa genotypes were identified as potential risk factors for precocious puberty.

Potential mechanism linking ERβ RsaI polymorphism to pubertal onset: Pubertal initiation is driven by increased pulsatile secretion of gonadotropin-releasing hormone (GnRH) from the hypothalamus. Although our findings demonstrate a statistical association between the ERβ RsaI polymorphism and ICPP, it is essential to propose a biologically plausible mechanism. GnRH neurons primarily express estrogen receptor β (ERβ) rather than ERα (26). ERβ within GnRH neurons mediates estrogen feedback signals to the hypothalamic-pituitary-gonadal (HPG) axis (24, 27). The RsaI polymorphism (rs1256049) is located in the ligand-binding domain of ERβ (9).which may alter receptor conformation or affinity for estradiol. We hypothesize that the R allele or Rr genotype could reduce ERβ sensitivity to negative feedback by estradiol during late childhood, resulting in diminished suppression of GnRH pulse generator activity. Consequently, premature activation of the HPG axis would occur, leading to early pubertal onset. This hypothesis is supported by animal studies: female ERβ knockout mice exhibit delayed puberty due to reduced pulsatile GnRH release (24), and loss-of-function ESR2 variants are associated with gonadal dysgenesis and HPG axis dysregulation (25, 27). Therefore, the RsaI polymorphism may represent a functional variant that modulates estrogen feedback sensitivity, thereby influencing pubertal timing. Future functional assays, such as luciferase reporter or estrogen-binding studies, are needed to directly test this mechanism.

This study specifically investigates the association between ERβ gene polymorphisms at the RsaI and AluI restriction sites and the risk of ICPP in girls affected with ICPP compared to healthy controls.Using polymerase chain reaction-restriction fragment length polymorphism (PCR-RFLP) methodology, the research aims to delineate differences in genotype and allele frequencies to clarify the genetic contribution of ERβ variants to disease susceptibility. The findings indicate a significant correlation between the RsaI polymorphism and increased risk of ICPP, suggesting that the R allele may act as a genetic susceptibility factor. These results provide novel insights into the molecular genetic landscape of ICPP. They also lay the groundwork for subsequent discussions on the functional implications of ERβ polymorphisms in estrogen signaling pathways, neuroendocrine regulation, and cellular mechanisms pertinent to early pubertal onset. The study’s outcomes thus hold promise for enhancing molecular risk prediction and informing individualized clinical management of ICPP.

One limitation of this study is that the sample size, while comparable to or larger than many previous genetic studies on ICPP (e.g.100 cases per group), may still be insufficient to detect very weak effects or rare haplotypes. Moreover, the homogeneity of our study population, all drawn from a single medical center, raises concerns regarding the external validity of our findings, as geographic and ethnic variations could influence the generalizability of the results. Additionally, the absence of functional assays to directly verify the biological implications of ERβ gene polymorphisms represents a significant gap, potentially undermining the depth of our conclusions regarding the mechanistic roles of these polymorphisms in ICPP pathogenesis.

## 5 Conclusion

In summary, our research highlights a significant association between the RsaI polymorphism of the ERβ gene and the risk of idiopathic central precocious puberty (ICPP), indicating that the R allele may serve as a genetic susceptibility factor, particularly in girls with the Rr genotype. This study offers valuable insights for early risk prediction and personalized therapeutic strategies in ICPP. Future investigations should aim for multicenter, larger-scale studies to validate these associations and further elucidate the functional mechanisms of ERβ, as well as explore its clinical applicability in endocrine disorders.

## Data Availability

The original contributions presented in the study are included in the article/supplementary material. Further inquiries can be directed to the corresponding author/s.
